# Role of clonal lineage analysis via next-generation sequencing in identifying the origin of multiple cancers and guiding treatment options

**DOI:** 10.1093/jjco/hyaf222

**Published:** 2026-01-23

**Authors:** Rie Shimoyachi, Aya Takimoto, Taichi Yoshida, Koji Fukuda, Kazuhiro Shimazu, Daiki Taguchi, Naoaki Kodama, Tomohiro Matsumoto, Toshiki Wakabayashi, Kazuhiro Imai, Hiroshi Nanjyo, Hiroyuki Shibata

**Affiliations:** Department of Clinical Oncology, Graduate School of Medicine, Akita University, Hondo1-1-1, Akita 010-8543, Japan; Department of Clinical Oncology, Graduate School of Medicine, Akita University, Hondo1-1-1, Akita 010-8543, Japan; Department of Clinical Oncology, Graduate School of Medicine, Akita University, Hondo1-1-1, Akita 010-8543, Japan; Department of Clinical Oncology, Graduate School of Medicine, Akita University, Hondo1-1-1, Akita 010-8543, Japan; Department of Clinical Oncology, Graduate School of Medicine, Akita University, Hondo1-1-1, Akita 010-8543, Japan; Department of Clinical Oncology, Graduate School of Medicine, Akita University, Hondo1-1-1, Akita 010-8543, Japan; Department of Clinical Oncology, Graduate School of Medicine, Akita University, Hondo1-1-1, Akita 010-8543, Japan; Department of Clinical Oncology, Graduate School of Medicine, Akita University, Hondo1-1-1, Akita 010-8543, Japan; Department of Surgery, Akita City Hospital, Kawamotomatsuoka-machi 4-30, 010-0933, Japan; Department of Thoracic Surgery, Akita University Graduate School of Medicine, Akita 010-8543, Japan; Department of Pathology, Akita University Hospital, Hondo1-1-1, Akita 010-8543, Japan; Department of Clinical Oncology, Graduate School of Medicine, Akita University, Hondo1-1-1, Akita 010-8543, Japan; Center for Cancer Registry and Information Services, Akita University Hospital, Hondo1-1-1, Akita 010-8543, Japan

**Keywords:** hereditary breast and ovarian cancer syndrome, next-generation sequencing, multiple primary cancer, pancreatic cancer, lung adenocarcinoma

## Abstract

Multiple cancers occur in the same individual, such as hereditary breast and ovarian cancer (HBOC) syndrome and Lynch syndrome. Here, we report a patient with HBOC syndrome who developed four different cancer types (pancreatic cancer, right lung adenocarcinoma, prostate cancer, and left lung adenocarcinoma) within a relatively short period of 6.5 years. In HBOC syndrome, the lung adenocarcinoma is rare, and the tumors were initially suspected to be lung metastases from pancreatic cancer, respectively. The pathological analysis results in each of the three lesions were inconsistent. A whole-exome analysis was performed on all three tumors using next-generation sequencing (NGS). The results showed that many of the deletion mutations found in pancreatic cancer were not present in other lung tumors. Homologous recombination is required for the repair of deletion mutations, but this function is impaired in HBOC syndrome. Deletions occurring in the primary tumor are irreversible and should be inherited in metastatic lesions. Therefore, we hypothesized that these three cancers arose independently, that each lung tumor was a primary tumor rather than a metastasis of pancreatic cancer, and that their resection would be curative. This assumption was reasonable, as no new lesions were observed in a 10-year follow-up study since the onset of pancreatic cancer. Tracking genetic traits using NGS helps understand the origins and progression of malignant tumors.

## Teaser

**Mini-abstract** Multiple cancers are likely to occur in conditions such as HBOC syndrome, which has a defect in gene repair function. In such cases, it can be difficult to determine whether each cancer is primary or a metastasis from an existing cancer. We experienced a case in which bilateral lung tumors developed following pancreatic cancer in a patient with HBOC syndrome. Initially, metastasis from the pancreatic cancer was suspected, but most deletion mutations found in the pancreatic cancer by NGS analysis were not found in the lung tumors. These were determined to be de novo early-stage lung cancers. The lung tumors were surgically removed. The patient has survived for 10 years since the onset of the first pancreatic cancer, and it can be said that lineage analysis by NGS correctly demonstrated that the lung tumors were not metastases.

## Introduction

The incidence of multiple primary cancers ranges between 2% and 6.3%, and the occurrence of four or more independent primary malignancies is estimated to be <0.1% [1, 2]. The frequency of multiple cancers is particularly high in familial tumors [3].

Hereditary breast and ovarian cancer (HBOC) syndrome is an autosomal dominant disorder caused by germline mutations in *BRCA* genes, primarily *BRCA1* and *BRCA2*, leading to an increased risk for breast and ovarian cancers, as well as pancreatic, prostate, and other malignancies [4]. Mutations in *BRCA1/2* not only predispose carriers to these cancers but also increase the overall likelihood of developing multiple primary tumors, including pancreatic, prostate, biliary tract, esophageal, and gastric cancers.

The BRCA1 protein consists of 1863 amino acids involved in DNA repair and checkpoint control [4]. BRCA1 functions in maintaining genomic stability through homologous recombination (HR) repair and cell cycle modulation and plays a role in apoptosis [4].

The BRCA2 protein consists of 3418 amino acids, and its primary role is in the HR repair of DNA double-strand breaks [4].

In western countries, *BRCA1/2* mutation carriers have a cumulative pancreatic cancer risk of 2%–5% [5]. Japanese data show an even higher risk of 16.0% for *BRCA1* carriers and 13.7% for *BRCA2* carriers by age 85 [6]. Men with HBOC syndrome, particularly those with *BRCA2* pathogenic variants, face a 2- to 6-fold increased risk of developing prostate cancer [7].

A Japanese cohort reported a 27% cumulative prostate cancer risk by age 80 among *BRCA2* carriers [6]. These prostate cancers are often more aggressive, with higher rates of lymphatic and distant metastasis as well as poorer prognosis [7].

Genetic testing plays a critical role in early diagnosis and therapeutic decision-making in various cancer cases.

Next-generation sequencing (NGS) is a cost-effective tool that can quickly and accurately sequence millions of DNA fragments, providing detailed information about the genome structure, genetic variations, and other key genomic features [8].

Advances in genetic technology have led to the increasing use of NGS, allowing for the simultaneous testing of multiple genes.

We experienced a case of multiple cancers in a patient with HBOC syndrome and a germline mutation in the *BRCA2* gene. In this case, curative treatment, resection and intensity-modulated radiation therapy (IMRT), was performed for early-stage pancreatic and prostate cancers, but lung tumors developed in the left and right lungs, in that order, 5 and 6.5 years later, respectively. In these cases, diagnosing whether multiple tumors occurring in the same patient are primary or metastatic is often difficult.

NGS was utilized to distinguish between primary and metastatic tumors. The case highlights the clinical utility of the NGS-based genomic analysis in the diagnostic and therapeutic decision-making of patients with hereditary cancer syndromes.

## Case report

**Figure 1:**
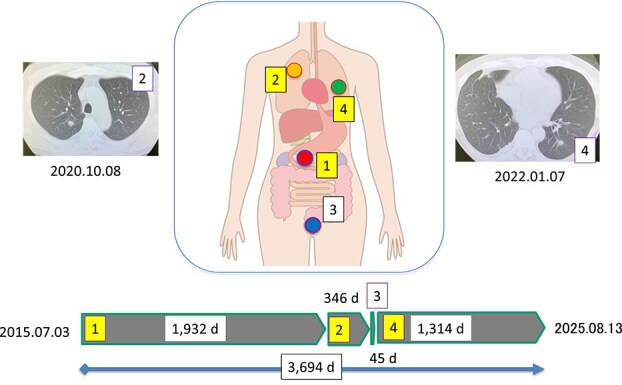
Overview of the patient’s clinical course; the first onset of pancreatic cancer (red circles) at 03 July 2015.; right lung cancer (orange circle), prostate cancer (blue circle), and left lung cancer (green circle) occurred sequentially; the number indicates the order of onset. The insets are the CT images of two lung cancers. “d” indicates days.

The patient was a 59-year-old male at the time of initial presentation. As outlined in Fig. 1, he underwent pylorus-preserving pancreaticoduodenectomy for pancreatic head cancer in July 2015 (pathological staging: pT3N2M0), followed by 1 year of adjuvant chemotherapy with S-1 (Fig. 1). In October 20XX + 5, a solitary adenocarcinoma was identified in the right lung, and right upper lobectomy with ND2a-1 lymph node dissection was performed (Fig. 1).

In September 20XX + 6, he was diagnosed with prostate cancer (cT2cN0M0) and received IMRT. A single administration of leuprorelin was given but not continued (Fig. 1). In November of the same year, a solitary lesion in the left lung was detected, which was initially referred to our department as a suspected pulmonary metastasis from pancreatic cancer (Fig. 1).

The laboratory findings at the time of referral were as follows: white blood cell: 5100/μl, neutrophils: 3400/μl, hemoglobin: 13.0 g/dl, platelets: 18.7 × 10^4^/μl, albumin: 4.2 g/dl, aspartate aminotransferase: 25 U/l, alanine transaminase: 22 U/l, total bilirubin: 0.8 mg/dl, creatinine: 0.86 mg/dl, sodium: 140 mEq/l, potassium: 4.3 mEq/l, corrected calcium: 9.8 mg/dl, chloride: 104 mEq/l, C-reactive protein: 0.07 mg/dl, carcinoembryonic antigen: 2.3 ng/ml, CA19–9: 21.8 U/ml, and duodenal pancreatic cancer antigen 2 <25 U/ml. The performance status was 0, and no abnormal physical findings were observed.

**Figure 2:**
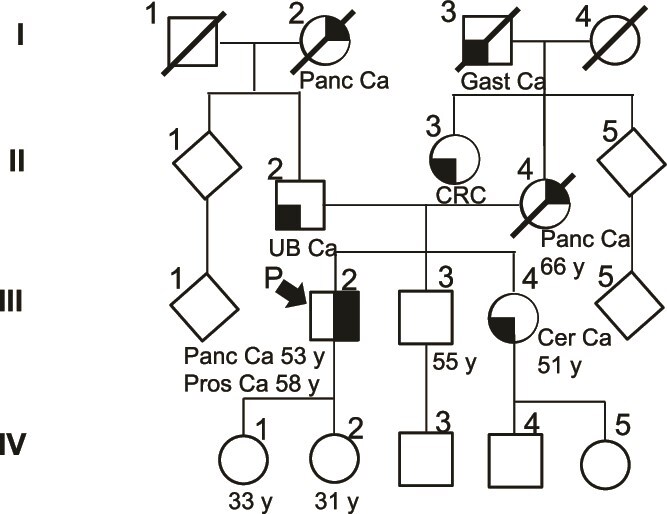
Patient’s family tree; Panc Ca: pancreatic cancer; Gast Ca: gastric cancer; UB Ca: urinary bladder cancer; CRC: colorectal cancer; Cer Ca: cervical cancer; “P”: proband; “y”: year.

Based on the abovementioned medical history, HBOC syndrome was suspected. His family history revealed pancreatic cancer in the paternal grandmother (I-2, Fig. 2), urinary bladder cancer in the father (II-2, Fig. 2), gastric cancer in the maternal grandfather (I-3, Fig. 2), pancreatic cancer in the mother (II-4, Fig. 2), and colorectal cancer in the maternal aunt (II-3, Fig. 2). The patient’s younger sister (III-4, Fig. 2) had cervical cancer in her 40s. The OncoGuide™ NCC Oncopanel System (Sysmex, Kobe, Japan) of the right lung tumor revealed NM_000059.4(BRCA2):c.4646AAG>A (p.E1550 fs*4) (allele frequency: 0.415), leading to a diagnosis of the germline mutation and confirming HBOC syndrome.

This variant is pathological, resulting in loss of function due to disruption of DNA-binding sites, Rad51-binding sites, and nuclear localization signals of BRCA2 [9, 10].

**Figure 3:**
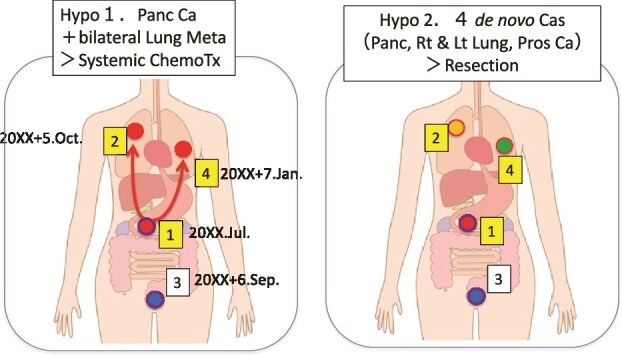
Hypothesis on the origin of multiple cancers in the patient; hypothesis (Hypo) 1 shows that two lung cancers (red circles) are metastases from the pancreatic cancer (red circle with a blue line); the blue circle indicates prostate cancer; the numbers indicate the order of the onset date. In this case, systemic chemotherapy (Systemic ChemoTx) is recommended; Hypo 2 shows that four de novo cancers (4 de novo Cas), namely, pancreatic cancer (red circle), right lung cancer (orange circle), prostate cancer (blue circle), and left lung cancer (green circle), arose independently. In this case, surgical resection is recommended.

At first, he was referred to our department for treating his left lung metastasis of pancreatic cancer. Accordingly, chemotherapy against metastatic pancreatic cancer with gemcitabine plus nab-paclitaxel was initiated. However, because it was a solitary lung mass, it was also considered to be a primary lung cancer (Fig. 3). In January 20XX + 7, segmentectomy was performed for the left lung tumor, with the patient subsequently receiving six cycles of adjuvant carboplatin plus paclitaxel for lung cancer.

We then conducted immunohistochemistry (IHC) to clarify the relationship between the pancreatic cancer and two lung tumors (i.e. whether primary tumor or lung metastases). IHC was analyzed by using an anti-CK 7 antibody (clone SP52, Roche Diagnostics K.K. Tokyo, Japan), an anti-CK 20 antibody (clone SP33, Roche Diagnostics K.K.), an anti-CA19–9 antibody (clone 121SLE, Roche Diagnostics K.K.), anti-CEA antibody (DAKO clone II-7, Agilent Technologies Japan, Ltd. Tokyo, Japan), an anti-MUC1 antibody (clone H23, Roche), and an anti-MUC5AC antibody (clone MRQ-19, Roche Diagnostics K.K.).

**Figure 4:**
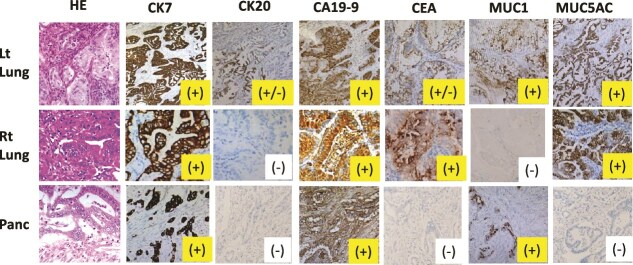
Immunohistochemistry results; Lt Lung: left lung tumor; Rt Lung: right lung tumor; Panc: pancreatic cancer; “(+)” indicates immunoreactivity, and “(−)” indicates no immunoreactivity.

Figure 4 summarizes the IHC profiles of the pancreatic and bilateral lung tumors. The profiles differed among the three tumors, supporting the possibility that the two lung tumors were independent from the pancreatic cancer. Figure 3 shows the two proposed diagnoses based on the patient’s presentation. The diagnosis of de novo primary lung cancers was immature. Although no recurrence was observed, the possibility of pulmonary metastasis from pancreatic cancer could not be entirely excluded. Given the presumed platinum sensitivity of pancreatic cancer, olaparib, a poly-ADP ribose polymerase (PARP) inhibitor, was proposed as a therapeutic option.

With the patient’s permission, the resected pancreatic cancer and two lung cancers were analyzed using NGS with full exome sequencing. The NGS of the three tumors was outsourced to Chemical Dojin Co., Ltd. (Kumamoto, Japan) using Illumina NovaSeq 6000. The reliability of the obtained data was high, with Q30 scores exceeding 90%, a read error rate below 0.1%, coverage ranging from 91.7% to 99.7%, and depth between 12.8 and 55.3. These metrics provided sufficient resolution for a comprehensive genomic evaluation.

We examined the results of the single-nucleotide variant (SNV) analysis of the *KRAS* and *EGFR* genes, of which mutations are frequently found in pancreatic cancer and lung adenocarcinoma. In pancreatic cancer, no SNVs were found in the *KRAS* gene exons, but four SNVs were found in the introns. These variants were not found in bilateral lung tumors. When comparing the left and right lung tumors of the 10 SNVs in the exon/intron, including the 3′untranslated region (UTR), of the *KRAS* gene found in the right lung tumor, one SNV in the 3′UTR and four SNVs in the introns were found in both tumors; however, one exon variant (A>G, rs1137282) and four intronic SNVs in the right lung tumor were not found in the left lung tumor. This exon variant (A>G, rs1137282) had a quality value for the variant as high as 572.77. From these points, considering the left lung tumor as intrapulmonary metastasis from the earlier right lung tumor is inconsistent. Focusing on the *EGFR* gene, three SNVs in the exons and three intronic SNVs were commonly found in the *EGFR* gene in pancreatic cancers and right lung tumors. We did not find five intronic SNVs in the right lung tumors. The quality values for the variant for these five SNVs were relatively low, but those scores are as low as 80. A common filtering criterion in the literature is that the quality score of the mutation is greater than 20. The analysis of the *EGFR* gene SNVs cannot completely rule out a lineage from pancreatic cancer to the right lung tumors. Furthermore, the right lung tumors contained 36 exon/intron SNVs, including the 3′UTR of the *EGFR* gene. The number of SNVs was reduced to 21 in the left lung tumors, which would be inconsistent if intrapulmonary metastasis to the left had occurred from the earlier right lung tumor.

**Figure 5:**
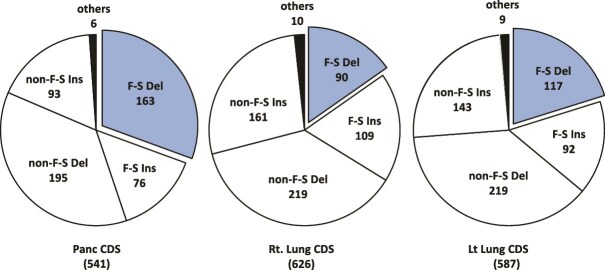
Frequency of the insertion/deletion of the coding sequence. “F-S Del,” “F-S Ins,” “non-F-S Del,” and “non-F-S Ins” indicate frameshift deletion, frameshift insertion, non-frameshift deletion, and non-frameshift insertion, respectively; “non-F-S Del,” means deletion but not resulting in frameshift. “non-F-S Ins” means insertion but not resulting in frameshift; “CDS” indicates the coding sequence, and the numbers are the frequency; Panc: pancreatic cancer; Rt Lung: right lung tumor; and Lt Lung: left lung tumor.

Next, we focused herein on the insertions and deletions (indels). Figure 5 presents a summary of the indels in the coding sequences. The total number of indels was 541 in pancreatic cancer, 626 in the right lung tumors, and 587 in the left lung tumors, which increased in the lung tumors. When looking only at frameshift deletions, the number was 163 in pancreatic cancer but decreased to 90 and 117 in the right and left lung tumors, respectively (Fig. 5). Deletion mutations that occurred in the primary tumor are unlikely to recur in the metastatic tumor.

We focused on the *KRAS* and *EGFR* genes and examined the indels in these genes. The three deletion mutations in the *KRAS* gene found in pancreatic cancer were not found in either of the two lung tumors (Table 1). If pancreatic cancer is the primary tumor, these deletion mutations should also be inherited in the lung metastases. Similarly, in the *EGFR* gene, six of the seven deletion mutations found in the pancreatic cancer were not inherited in either of the two lung tumors. None of the three deletion mutations found in the right lung tumor that developed first were found in the left lung tumor, which would be contradictory if the right lung cancer had metastasized to the left. From the above, each of the three tumors is believed to be an independent carcinoma that developed de novo.

**Table 1:** Deletion variants at the *KRAS* and *EGFR* gene loci in the pancreatic, right, and left lung cancers.

Gene, Chr.	Position	Pancreas cancer	Right lung tumor	Left lung tumor
*KRAS,* 12	25214387^a^	–	–	CTT>T
*KRAS,* 12	25218945^a^	GTT>T	–	–
*KRAS,* 12	25327902	–	–	C(AAAT)_5_>C
*KRAS,* 12	25362243	T(A)_18_>T	–	–
*KRAS,* 12	25398502	–	–	ATTTG>A
*KRAS,* 12	25426004	GCC>G	–	–
*KRAS,* 12	25754999	–	–	CAA>C
*KRAS,* 12	25768436	–	–	CTTCT>C
*KRAS,* 12	25785715	–	–	TG>T
*KRAS,* 12	25850371	–	–	AT>A
*KRAS,* 12	25928334	–	–	(A)_4_C>A
*EGFR*, 7	55035510^a^	TA>T	TA>T	–
*EGFR*, 7	55089939^a^	–	–	C(TTAT)_3_>C
*EGFR*, 7	55205665	CA>C	–	–
*EGFR*, 7	55260330	–	TCCG>T	–
*EGFR*, 7	55326185	–	TC>T	–
*EGFR*, 7	55333927	AAAGG>A	–	–
*EGFR*, 7	55338825	–	–	C(A)_6_>C
*EGFR*, 7	55340271	–	–	ATTT>A
*EGFR*, 7	55341592	–	–	CG>C
*EGFR*, 7	55706979	AAAGG>A	–	–
*EGFR*, 7	55763549	TA>T	–	–
*EGFR*, 7	55779572	AT(TTC)_5_TT(TTC)_2_>A	–	–
*EGFR*, 7	55788474	CCT>C	–	–

The left side of > indicates the reference sequence, while the right side is the result of deletion (e.g. “GTT>T” indicates that “GT” is deleted from the “GTT” sequence, and “T” is left behind). The subscript indicates the number of repetitions [e.g. “(AAAT)5” indicates a five-time repeat of the “(AAAT)” sequence].

^a^Indicates intronic variant. The others are intergenic variants. The position is the position of the variant on chromosomes. Chr: chromosome; –: not available.

Homologous recombination repair deficiency (HRRD) leads to a variety of structural rearrangements, including insertions, deletions, abnormal copy number, and inter-chromosomal linkages. On the other hand, mismatch repair deficiency (MMRD) leads to the accumulation of frameshift mutations and is associated with the expression of potent neoantigens. This genomic instability can lead to the accumulation of various mutations, including frameshift mutations. Numerous frameshift mutations were found in this case. It was generally believed that frameshift mutations accumulate due to MMRD and are incompatible with HRRD, which causes genomic instability [11]. However, in recent years, the existence of proteins such as PMS2 and MSH2 that link MMRD and HRRD has become known, demonstrating a relationship between the two [12]. Therefore, we examined mutations in MMR-related genes in each tumor. No somatic mutations that could possibly lead to MMRD were found in the three cancer types in this case (Supplementary Fig. S1).

Analysis of the *BRCA2* gene locus, including full-exome sequencing, revealed 10 SNVs in the right lung cancer and nine SNVs in the left lung cancer in the region from the 5′ UTR to exon 10 of the *BRCA2* gene (data not shown). Of these, six were common to both lung cancers. All but one in the left lung cancer were assigned ID numbers and were previously reported SNVs. These are thought to be genetic polymorphisms rather than pathogenic mutations. However, these SNVs were absent in pancreatic cancer. This indicates the presence of a large deletion in the *BRCA2* gene from the 5′ UTR to exon 10 in pancreatic cancer. This deletion was thought to be a homozygous deletion including the normal allele. While it is speculated that BRCA2 inactivation occurs in both lung cancers due to loss of the normal wild-type allele, the above-mentioned mechanism is suggested to be responsible for BRCA2 inactivation in pancreatic cancer.

Olaparib is still administrated for 3 years without any adverse events. The patient survives with a cancer-free status over 10 years since the first onset of pancreas cancer (Fig. 1). Therefore, we conclude that our assumption is correct.

## Discussion

This case presents an extraordinarily rare instance of quadruple primary cancers—initial pancreatic head cancer, followed by right lung adenocarcinoma, prostate cancer, and left lung adenocarcinoma. With advances in diagnostic technology and increased life expectancy, reports of such cases have been rising, especially in patients with underlying genetic predispositions or genomic instability.

The variant allele frequency analysis is effective in identifying the clonal lineage [13] and determining whether multiple tumors occurring in a patient represent a “primary and its metastatic tumor-relationship” or are independent. Common genetic variants are found in both primary and metastatic tumors, but additional variants are also added as the cancer progresses.

Deletions are not considered to be restored during tumor progression. Specifically, deletions present in the primary tumor are expected to be inherited in metastatic tumors. In patients with HBOC syndrome, where HR is deficient, such deletions cannot be repaired. Among various HR-deficient cell lines, short deletions were most frequently observed in *BRCA2*-deficient (*BRCA2*^−/−^) cell lines, with over 30 deletions per genome compared to fewer than five in the wild-type cells [14].

Taken together, this case highlights the value of comprehensive genomic profiling using NGS in the diagnosis and management of patients with suspected multiple primary cancers, providing a notable example of how genomic diagnosis can support clinical decision-making and individualize cancer treatment strategies.

BRCA2 p.E1550 fs*4 has not been reported in ClinVar database, but the allele frequency of the similar variant, BRCA2 p.E1550X, was 0.00001 in the ClinVar database [15]. According to the jMorp database, the allele frequency of BRCA2 p.E1550X in the general Japanese population is 0.000003–0.000007 [16]. Additionally, according to the Breast Cancer Information Core database, a total of 14 914 BRCA2 mutations have been registered, but BRCA2 p.E1550X has only occurred twice [17]. The top 20 BRCA2 mutations have been recorded at least 128 times, making BRCA2 p.E1550X a rare pathogenic mutation. Two cases of BRCA2 p. E1550fs*4 have been reported in the Tohoku region of Japan; one is this case, but the other was a case of colorectal cancer [10].

Individuals with a *BRCA2* mutation had a higher incidence of pancreatic cancer than the general population [standardized incidence ratio (SIR) 21.745, *P* < .001] [18]. Prostate cancer was most significantly associated with male patients carrying a *BRCA2* mutation (SIR 4.890, *P* = .002) [18]. Other than breast, ovarian, pancreas, and prostate cancers, individuals with *BRCA2* mutations also had cervical (6 out of 406 cases) and lung cancers (5 out of 406 cases) [18]. These values were the highest and ranked second. While the SIR of cervical cancer was 4.41 (*P* = .006), that of lung cancer was 4.867 (*P* = .929) [18]. The younger sister of the patient suffered from cervical cancer. Although the lung cancer incidence in HBOC syndrome is extremely low, some reports have been made. A total of 20 pathogenic *BRCA1* (0.22%) and 66 pathogenic *BRCA2* (0.73%) germline mutations were identified among 9010 Chinese patients with non-small cell lung cancer (NSCLC) [19]. In China, the pathogenic germline mutation frequency of *BRCA2* was 0.79% in lung cancer patients [20]. Pathogenic *BRCA1/2* mutations were found in 2.1% in French NSCLC [21]. As reported, patients of lung cancer with the *BRCA2* germline mutations might benefit from PARP inhibitor treatment [22].

In recent years, several reports have been published regarding the association between lung cancer and germline mutations in DNA repair genes. Sorscher *et al*. found that of 7788 lung cancer patients (2015–22) who underwent germline genetic testing, 14.9% (1161 of 7788 cases) had pathogenic germline mutations, with significantly increased rates in BRCA2 (2.8%), CHEK2 (2.1%), ataxia telangiectasia mutated (ATM) (1.9%), BRCA1 (1.2%), and mismatch repair genes (1.2%) compared to the control group [23]. A similar report was made by Shevach *et al*. in a cohort study of 183 627 patients, finding that lung cancer was significantly associated with rare pathogenic variants in ATM [odds ratio (OR) 1.94, 95% confidence interval (CI): 1.14–3.07] and BRCA2 (OR 2.78, 95% CI: 1.73–4.23) [24]. Furthermore, Yang *et al*. reported that 8.1% (9/111) of patients with multiple primary lung cancers had germline mutations in genes involved in DNA damage repair, with the most common genes being the BRCA1/2 genes (1.8%) and the Werner syndrome ATP-dependent helicase (WRN) gene (1.8%) [25]. Germline mutations in genes involved in DNA damage repair were also found in 54/625 (8.6%) patients with solitary lung cancer.

As mentioned above, among DNA damage repair genes associated with lung cancer, the BRCA2 gene has a high frequency of pathogenic germline mutations. Also, according to Prassas *et al*., among carriers of BRCA2 pathogenic variants (*N* = 1629), the hazard ratio (HR, 95% CI) for breast cancer (female) was 5.49 (4.82–6.25), for breast cancer (male) was 13.99 (5.90–33.16), for ovarian cancer was 11.05 (8.65–14.11), and for pancreatic cancer was 3.75 (2.40–5.86). The HR for lung cancer was 2.76 (2.05–3.72). Lung cancer is typically considered to have non-canonical HBOC malignancies, but its HR has been shown to be higher than that of prostate cancer, at 2.68 (2.20–3.27) [26].

Synthetic lethality is induced by PARP inhibition in *BRCA*-mutated cancer cells [27]. Maintenance treatment with olaparib could prolong the progression-free survival of patients with germline *BRCA*-mutated pancreatic cancer [28].

The genetic testing for HBOC syndrome is typically first performed on affected individuals in a family. Cascade testing can be offered to at-risk relatives if a pathogenic *BRCA* mutation is identified. The patient has two daughters in their 30s (IV-1, 2, Fig. 2), and their genetic testing has been recommended to our patient.

## Conclusion

In patients with HBOC syndrome, vigilance is required for both breast and ovarian cancers and for potentially developing pancreatic and prostate cancers. NGS is a valuable tool for clonal lineage analysis in cases of multiple malignancies, e.g. bilateral lung tumors in our case, offering significant aid in treatment planning and therapeutic decision-making.

## Supplementary Material

Sup_Fig_hyaf222

Supplemental_Figure_Legend_hyaf222

## Data Availability

Ethics approval and consent to participate. Written informed consent was obtained from the patient described in this report. The examination of genes responsible for familial cancers was approved by the Ethical Committee of Akita University (Approval No. 1191).
