# Supplementary figures and images for: Role of clonal lineage analysis via next-generation sequencing in identifying the origin of multiple cancers and guiding treatment options
Source: Jpn J Clin Oncol. 2026 Jan 23;56(4):489–95. doi: 10.1093/jjco/hyaf222 (PMC13070523; doi:10.1093/jjco/hyaf222)

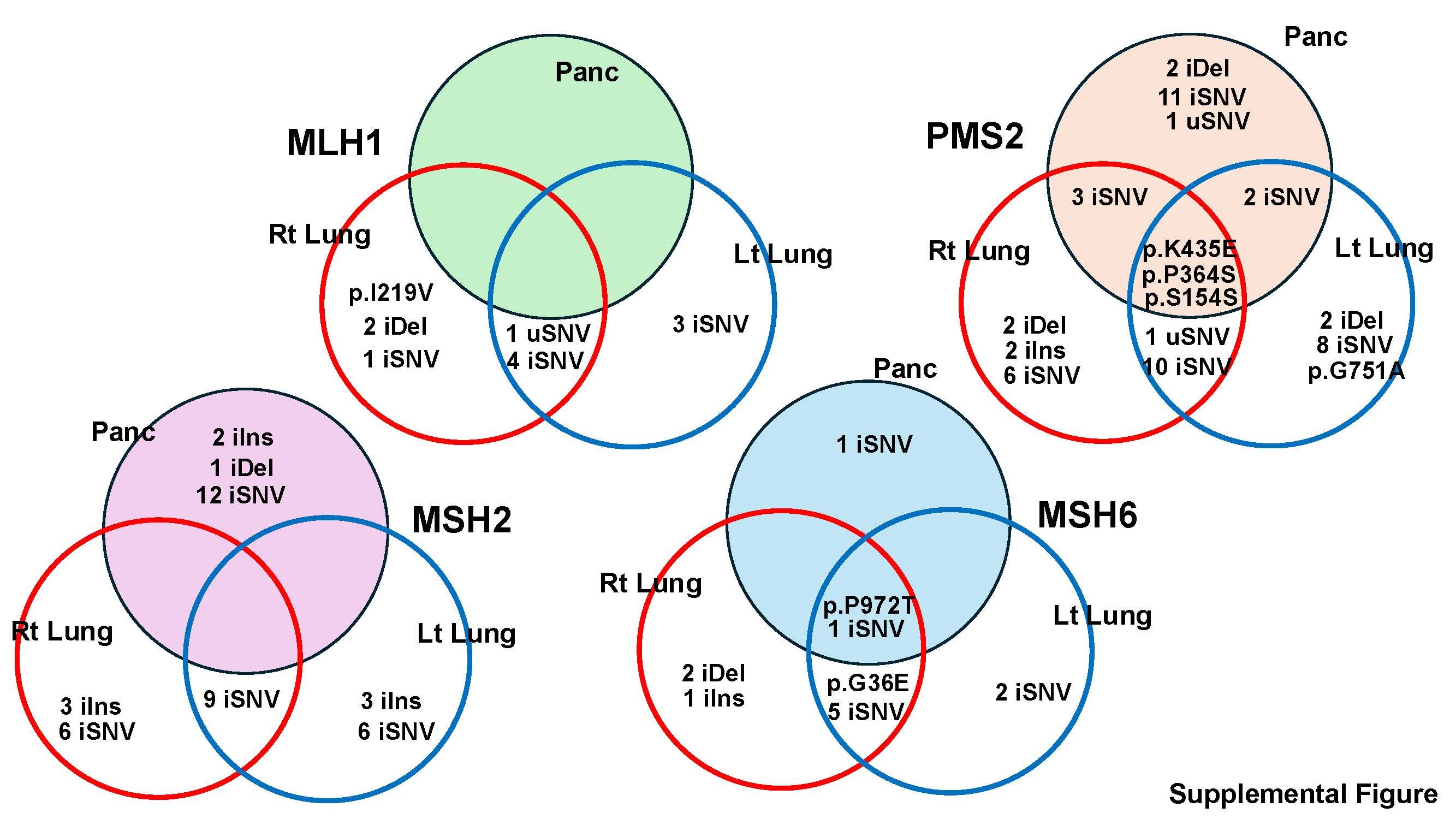

Supplement: Sup_Fig_hyaf222 [file sup_fig_hyaf222.jpeg]
