# Supplementary material for: Role of clonal lineage analysis via next-generation sequencing in identifying the origin of multiple cancers and guiding treatment options
Source: Jpn J Clin Oncol. 2026 Jan 23;56(4):489–95. doi: 10.1093/jjco/hyaf222 (PMC13070523; doi:10.1093/jjco/hyaf222)
Supplement: Supplemental_Figure_Legend_hyaf222 [file supplemental_figure_legend_hyaf222.docx]

Supplemental Figure Legend

Supplemental Figure. Gene alteration in mismatch repair genes

Numbers of gene alterations in mismatch repair genes including *MLH1*, *MSH2*, *MSH6* and *PMS2* genes in pancreas (Panc), right lung (Rt Lung) and left lung (Lt Lung) cancers. iDel and iIns indicate intronic deletion and insertion, respectively. iSNV and uSNV indicate single nucleotide variant (SNV) in intron and untranslated region, respectively.

In the *MLH1* gene, MLH1 p.I219V has not been reported in ClinVar database. None of these alterations have been reported to be pathogenic.

In the *MSH2* gene, none of these alterations have been reported to be pathogenic.

In the *MSH6* gene, a missense mutation (MSH6.p.G36E) and a synonymous mutation (MSH6.p.T972T) were found in an exon in right lung cancer. In pancreatic cancer, MSH6.p.T972T was found in an exon. In left lung cancer, MSH6.p.G36E and MSH6.p.T972T) were found in exons. MSH6.p.G36E has not been reported in ClinVar database. None of the above mutations have been reported to be pathogenic.

In the *PMS2* gene, two missense mutations (PMS2 p.K435E, PMS2 p.P364S) and one synonymous mutation (PMS2 p.S154S) were found in the right lung cancer. The same two missense mutations and one synonymous mutation were found in the exons of pancreatic cancer. The same two missense mutations and one synonymous mutation were also found in left lung cancer. One additional missense mutation (PMS2 p.G751A) was found. Three mutations, PMS2 p.K435E, PMS2 p.P364S, and PMS2 p.S154S, were commonly observed between left and right lung cancers and pancreatic cancers. PMS2 p.K435E was benign in ClinVar database, and PMS2 p.P364S had not been reported.
